# Bilateral neuroretinitis and anterior uveitis following ipilimumab treatment for metastatic melanoma

**DOI:** 10.1186/s12348-016-0082-3

**Published:** 2016-05-10

**Authors:** Laura Hahn, Kathryn L. Pepple

**Affiliations:** Department of Ophthalmology, University of Washington School of Medicine, Seattle, WA USA

**Keywords:** Ipilimumab, Uveitis, Neuroretinitis

## Abstract

**Background:**

This report presents a novel case of bilateral neuroretinitis and anterior uveitis in a patient receiving ipilimumab treatment for metastatic cutaneous melanoma and summarizes the literature regarding treatment options for patients with ipilimumab-related ocular immune-related adverse events.

**Findings:**

The medical chart was reviewed, a literature search was performed, and the results were summarized. For the case presented here, in addition to discontinuation of ipilimumab, combined topical and oral corticosteroid therapy provided visual recovery and resolution of ocular inflammation over 2 months. Review of the literature identified that the majority of reported patients required treatment with oral corticosteroids for control of ocular and periocular inflammation.

**Conclusions:**

Ipilimumab-induced ocular inflammation is a rare adverse immune event. This case, in conjunction with the current literature, suggests that in the setting of severe ocular inflammation, treatment with both topical and oral corticosteroids are typically required for management and preservation of good visual function.

## Findings

### Introduction

Ipilimumab is a monoclonal antibody directed against cytotoxic T-lymphocyte antigen 4 (CTLA4), a checkpoint molecule present on the surface of activated T-lymphocytes [1]. CTLA4 negatively regulates T-lymphocyte proliferation and activation, and inhibitors of this molecule are approved by the Food and Drug Administration (FDA) as a treatment for metastatic melanoma [2]. Treatment with ipilimumab causes tumor regression as well as immune-related adverse events (irAEs) in almost two thirds of patients. The most common irAEs include dermatitis, enterocolitis, and hypophysitis and are dose related [3]. Management of these toxicities depends on the severity and can be stratified into grades. In patients with clinically significant (stage 3 or 4) or severe irAEs, ipilimumab therapy is typically discontinued and systemic corticosteroids such as prednisone 1 to 2 mg/kg daily are given to control inflammation [4].

Ocular irAEs occur in less than 1 % of patients receiving ipilimumab treatment as compared to 44 % of patients reporting diarrhea or colitis as a side effect [5]. Case reports have described a range of ocular involvement including bilateral anterior uveitis, vitritis, and papillitis [5–7]; choroiditis and serous retinal detachments [8–10]; peripheral ulcerative keratitis (PUK) [7]; inflammatory orbitopathy [7, 11, 12]; choroidal neovascularization (CNV) [13]; uveitis with a myasthenia gravis-type syndrome [14]; and bilateral optic neuropathy [15]. This report describes neuroretinitis and anterior uveitis as a novel ipilimumab ocular irAE.

### Case report

A 44-year-old Hispanic male presented for evaluation of bilateral ocular inflammation in the setting of treatment with ipilimumab for metastatic cutaneous melanoma. Two months prior to presentation, he had received his third infusion with partial regression of his metastatic lesions. Subsequent to this dose, he began to experience systemic inflammatory side effects from therapy including rash, diarrhea, fever, and persistent rectal abscesses requiring treatment with a high-dose oral prednisone in addition to cessation of ipilimumab therapy. While on a tapering dose of oral prednisone (20-mg prednisone), he began to experience ocular symptoms including metamorphopsias in the right eye, scotoma in the left eye, bilateral eye pain, redness, and photophobia. One week later on examination with a comprehensive ophthalmologist, he was noted to have anterior chamber inflammation and bilateral optic nerve edema. An MRI of the brain was obtained, but it did not identify any mass lesions. Topical therapy with prednisolone acetate was initiated, and he was referred to the uveitis service for further evaluation.

Upon presentation, he denied eye pain or redness but reported persistent central scotoma in the left eye. Current therapy included bilateral prednisolone acetate 1 % four times daily, 0.2 % brimonidine tartrate-0.5 % timolol maleate ophthalmic solution twice daily, and 20 mg of oral prednisone daily. His other systemic autoimmune symptoms were well controlled. He did not have a history of prior autoimmune diseases and exposure to ticks or kittens and had no headache, pain with eye movement, or pulsatile tinnitus.

**Fig. 1:**
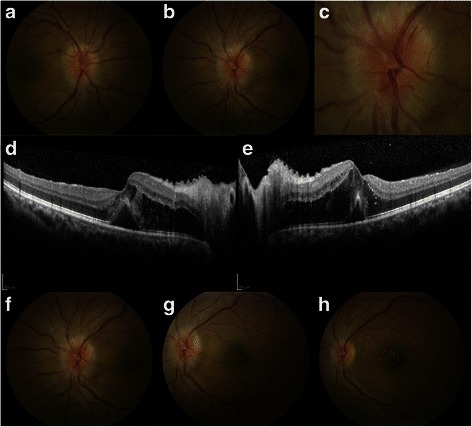
Ipilimumab-associated bilateral neuroretinitis. Presentation images showing disc edema with Paton’s lines of the **a** right and **b** left eyes. **c** Higher magnification image of the left eye reveals a splinter hemorrhage on the superior temporal nerve. Spectral domain OCT images of the **d** right and **e** left eyes reveal edema of the optic nerve and subfoveal and intraretinal fluid in both eyes. **d**–**h** Time course of images of the left eye from presentation **f** to 1-week follow-up **g** to 1-month follow-up **h** showing resolution of optic nerve and macular edema with development of a macular star

On examination, his visual acuity was 20/40-1 in the right eye and 20/150 in the left eye. Intraocular pressures were normal. Slit lamp examination revealed pigment on the endothelium without keratic precipitates (KP) bilaterally as well as 1+ flare and 0.5+ cell in both the anterior chambers. Rare cell was noted immediately posterior to the lens without vitreous haze or posterior vitritis. Fundus examination showed bilateral optic nerve edema with Paton’s lines and macular edema (Fig. 1a, b, f). In the left eye, a splinter hemorrhage was noted on the superior disc margin (Fig. 1c). The vessels were perfused without sheathing or arteriovenous crossing changes. The peripheral retina was unremarkable. Optical coherence tomography (OCT) revealed significant bilateral cystic intraretinal and subfoveal fluid (Fig. 1d, e).

Quantiferon testing and syphilis serology were negative, and his oral prednisone was increased to 80 mg daily. Topical prednisolone acetate was decreased to three times daily, and 0.2 % brimonidine tartrate-0.5 % timolol maleate ophthalmic solution was continued in both eyes. At one-week follow-up, his vision was 20/60 in the right eye and 20/50 in the left eye. Fundus photos showed improving optic nerve edema bilaterally, decreased retinal folds, and the initial development of a macular star in both eyes (the left eye shown in Fig. 1g). Oral prednisone was tapered by 10 mg per week down to 10 mg daily, and the topical prednisolone acetate was tapered to once daily in both eyes. Over the course of the next 3 weeks, his vision steadily improved with resolution of the optic nerve and retinal edema and subsequent development of a macular star (Fig. 1h). Due to gastrointestinal upset while taking 10 mg of prednisone a day, the patient discontinued oral corticosteroids without additional tapering and experienced a flare of bilateral anterior uveitis which was treated with 0.05 % topical difluprednate. No recurrence of the neuroretinitis was noted. By the 2-month follow-up, vision in both eyes improved to 20/25 and his anterior chamber reaction stabilized at 1+ flare and rare cell while taking prednisolone acetate four times a day. The patient was then lost to follow-up.

### Discussion

Treatment of ipilimumab-associated ocular irAEs depends on the severity and location of inflammation and may be influenced by concurrent therapy for other systemic complications. In the largest case series of patients with ipilimumab-associated ocular irAEs [7], 6/7 patients were treated with oral corticosteroids alone or in conjunction with topical corticosteroids. Oral corticosteroid treatment was initiated specifically for control of ocular disease in the four patients with orbital inflammation. In an additional of two patients with anterior uveitis, systemic corticosteroids were initiated for control of other inflammatory complications [7]. Topical corticosteroid drops were added in addition to ongoing systemic treatment for two patients with anterior uveitis and as sole therapy for the patient with PUK. The authors also reviewed 15 previously published cases of ipilimumab-associated ocular irAEs. They found that 9 of the 15 cases were treated with systemic corticosteroids and 3 with topical corticosteroids alone. One patient with CNV was treated with intravitreal ranibizumab without corticosteroids [13], one patient with a myasthenia gravis-type syndrome was treated with plasmapheresis [14], and one patient with conjunctivitis was treated with topical lubrication [16]. Most patients also had ipilimumab therapy withheld. Twelve of the 15 patients had resolution of their inflammation. The authors concluded that ocular irAEs from ipilimumab resolve with corticosteroid treatment often without sequelae. Subsequent to this review, a case of bilateral optic neuropathy was described by Yeh and Francis that was treated with topical therapy alone. In this case, anterior uveitis developed after the third round of ipilimumab therapy. His inflammation then evolved into bilateral optic nerve edema with macular edema and decreased vision after the fourth round of ipilimumab therapy. Oral therapy was declined by the patient, and he was treated with topical prednisolone acetate every 2 hours. Anterior segment and optic nerve inflammation resolved after 3 months, but optic nerve pallor developed. Despite regaining 20/25 central vision at 6 months, peripheral visual field defects did not resolve, and a new paracentral scotoma developed [7]. Our patient had a similar presentation to this patient with anterior uveitis and bilateral neuroretinitis. However, he was treated with high-dose (1 mg/kg) oral corticosteroids in addition to topical therapy with rapid control of inflammation (1 month rather than 5 months) and restoration of 20/25 vision without optic nerve pallor. Ipilimumab therapy was also discontinued, which may have also played a role in his improved visual outcome.

Understanding the natural history and good response to corticosteroids of most ipilimumab ocular irAEs is important for management, as discontinuation of ipilimumab therapy is reserved for severe or life-threatening irAEs. Our case, in conjunction with the summary of previously published cases, suggests that ocular complications can be managed with corticosteroid therapy if other systemic irAEs do not require discontinuation of therapy. In the presence of posterior uveitis or significant sight-threatening inflammation, systemic corticosteroids should be considered and can provide good visual recovery. Unfortunately, in the majority of the reported cases of ocular irAEs, the patients also experienced other grade 3 or 4 systemic complications, necessitating ipilimumab discontinuation.

### Consent

Written informed consent was obtained from the patient for the publication of this report.

## Abbreviations

CTLA4: cytotoxic T-lymphocyte antigen 4
CNV: choroidal neovascularization
irAEs: immune-related adverse events
OCT: optical coherence tomography
PUK: peripheral ulcerative keratitis
